# The Evolving Threat of African Swine Fever in Asia

**DOI:** 10.3390/pathogens14121278

**Published:** 2025-12-12

**Authors:** Wen-Hung Wang, Arunee Thitithanyanont, Sheng-Fan Wang

**Affiliations:** 1 Center for Tropical Medicine and Infectious Disease, Kaohsiung Medical University, Kaohsiung 80708, Taiwan; bole0918@gmail.com; 2 School of Medicine, College of Medicine, National Sun Yat-Sen University, Kaohsiung 804201, Taiwan; 3 Department of Microbiology, Faculty of Science, Mahidol University, Bangkok 10400, Thailand; arunee.thi@mahidol.ac.th; 4 Department of Medical Laboratory Science and Biotechnology, Kaohsiung Medical University, Kaohsiung 80708, Taiwan; 5 M.Sc. Program in Tropical Medicine, College of Medicine, Kaohsiung Medical University, Kaohsiung 80708, Taiwan; 6 Department of Medical Research, Kaohsiung Medical University Hospital, Kaohsiung 80708, Taiwan

## 1. Introduction

Originally identified in Kenya in 1921, African swine fever (ASF) primarily remained a sub-Saharan concern for nearly a century [1]. However, its disastrous introduction into East Asia via China in 2018 fundamentally altered the global swine production landscape [2]. Over the past seven years, the region has undergone a profound epidemiological shift: what began as an acute, invading panzootic has transitioned into a state of chronic, deeply entrenched endemicity.

In retrospect, this transition was a predictable consequence of the virus’s formidable biological makeup. As the sole member of the *Asfarviridae* family, African swine fever virus (ASFV) is a genetically complex, large double-stranded DNA virus. Its resilience is derived from an intricate, multilayered icosahedral morphology, incorporating an internal lipid membrane and an outer envelope hijacked from the host cell [3], which confers extraordinary environmental tenacity. ASFV can withstand broad pH and temperature ranges and retain infectivity for months in contaminated fomites, feed, or soil, and even longer in frozen pork products [4].

**Figure 1:**
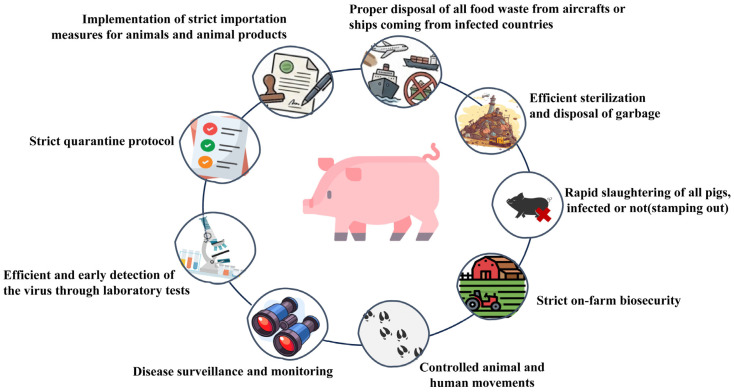
The diagram illustrates a multi-faceted approach to preventing viral introduction and limiting spread within swine populations. Key strategies include strict border control and quarantine protocols, management of food waste (swill) from international sources, rigorous on-farm biosecurity, active disease surveillance, and rapid response mechanisms such as stamping out.

This durability underpins complex transmission networks that are notoriously difficult to disrupt, necessitating a multilayered defense strategy. While direct contact is the primary route of transmission, indirect routes such as contaminated vehicles, equipment, personnel, and infectious swill feeding play a critical role [5]. Consequently, strict on-farm biosecurity, controlled animal and human movements, and the proper disposal and sterilization of food waste (particularly from international carriers) are essential in order to disrupt these networks (Figure 1). Furthermore, in specific ecological niches, sylvatic cycles involving *Ornithodoros* ticks act as long-term biological reservoirs [6]. Combined with a genome encoding numerous proteins that subvert host innate immunity, ASFV is uniquely suited for long-term persistence. Therefore, disease surveillance and efficient early detection through laboratory tests are indispensable (Figure 1). Asia is now facing the arduous reality of managing a resident, highly adaptable pathogen established across diverse ecological and production niches, where strict quarantine protocols and rapid stamping out remain the final lines of defense.

## 2. When the Last Barrier Breaks: The Breach of Taiwan

While widespread ASFV incursions have been reported across the region, for several years, Japan and Taiwan were the only East Asian nations to remain officially ASF-free. Japan has maintained historical freedom from the virus without any reported outbreaks. Regrettably, Taiwan lost its ASF-free status in October 2025 following a confirmed outbreak at a farm in Wuqi District. This detection, officially reported to the World Organisation for Animal Health (WOAH), represents a critical turning point for disease control in the region [7,8].

Taiwan was previously a regional anomaly, maintaining disease-free status despite close proximity to endemic neighbors and dense trade and travel links. This status was sustained through what was arguably one of the world’s most aggressive border enforcement regimes [9]; under a “zero-tolerance” policy, Taiwan implemented 100% X-ray screening of hand luggage from high-risk areas, immediate heavy fines (upwards of USD 6500) for travelers carrying undeclared pork products, and a decisive nationwide ban on the traditional practice of swill feeding to pigs. The successful incursion of the virus despite these measures underscores the difficulty of maintaining absolute exclusion against such a resilient pathogen in the long term.

## 3. From Virulent Waves to Silent Carriers

The antagonist in this ongoing crisis is not a static entity. In long-standing endemic zones, selective pressures on ASFV are shifting. While highly virulent genotype II strains remain dominant, the epidemiological picture has become increasingly blurred (Table 1). Historical experience indicates that in endemic regions, naturally attenuated strains may emerge over time, leading to chronic or subclinical infections that evade standard diagnostic and clinical surveillance protocols [10]. These less dramatic infections can quietly maintain transmission in systems where biosecurity is insufficient.

This natural evolution is now being aggressively accelerated by human intervention. Driven by economic pressure and desperation, the unlawful use of unapproved live-attenuated vaccines has yielded serious and sometimes disastrous consequences. Recent surveillance has detected vaccine-escape mutants and, most alarmingly, recombinant strains harboring genetic characteristics of both genotype I and II viruses [11].

Since 2021, novel genotype I/II recombinant strains have emerged in China and have subsequently spread to Northern Vietnam [12]. These viruses possess a unique and deceptive profile: they retain the lethal virulence of genotype II (including the *CD2v* gene, a key virulence factor in ASFV) while utilizing the genotype I backbone to evade protection from current genotype II-based vaccines [11]. Furthermore, these novel strains often manifest atypical or muted clinical signs, creating populations of “silent carriers” that facilitate viral movement through trade chains and production networks. This stealthy propagation undermines passive surveillance systems that rely heavily on mortality or classical clinical presentation as early warning signals.

## 4. The Illusion of a Quick Vaccine Fix

The increasing complexity of field strains underscores a critical danger: the industry’s hope for an immediate “vaccine savior.” The scientific obstacles to developing a safe, effective ASFV vaccine are immense. Natural infection does not reliably induce classical neutralizing antibodies, and correlates of protection remain poorly defined due to the virus’s complex immunomodulatory mechanisms [13]. While legitimate progress toward licensed live-attenuated vaccines has occurred in some nations [14], achieving a universally safe, genetically stable, and DIVA-compliant (Differentiating Infected from Vaccinated Animals) product suitable for widespread, largely unsupervised field use presents unprecedented challenges [13].

In this context, premature deployment of imperfect biologicals in an already complex epidemiological environment is not a solution; it acts as an accelerant for confusion and risk. Overreliance on a future technological fix also risks breeding complacency around the only defense currently proven effective against this environmentally stable virus: rigorous, consistently applied biosecurity along the entire production and value chain [15].

## 5. Conclusions

The consolidation of ASF endemicity across Asia serves as a stark reminder that, against such a biologically resilient pathogen, “zero-risk” zones are likely to be temporary. The region must transition from an “exclusion mindset” to preparing for a multi-decade course of disease management. The path forward necessitates a strategic realignment grounded in scientific reality. Governments must prioritize transparent genomic and epidemiological surveillance to track viral evolution and detect emergent variants. The industry must accept that modernization, compartmentalization, and rigorous biosecurity segregation are now baseline requirements for continuing production in an endemic environment. Until the complex immunological hurdles of ASFV are fully overcome to produce a truly safe, effective, and DIVA-compliant vaccine, biosecurity resilience will remain the primary form of protection between the swine industry and recurrent waves of devastating production losses.

## Figures and Tables

**Table 1:** ASFV status and genotype distribution in Asia (updated November 2025).

Country	Severity/Status	Circulating Genotype	Remarks
China	Widespread/Endemic	Genotype I/II Recombinant	Source of diverse viral strains including the highly virulent recombinant (I/II) variant.
Vietnam	Widespread	Genotype II Recombinant	Recombinant strains (I/II) confirmed in Northern provinces; poses high risk of regional spread.
Taiwan	Local Outbreak	Genotype I/II Recombinant	The first confirmed domestic ASF outbreak occurred in October 2025 at a large pig farm in Wuqi District. According to the Ministry of Agriculture (MOA), the outbreak-associated strain exhibits high similarity to strains currently found in China and Vietnam.
Philippines	Regional/Active	Genotype II	Persistence in multiple provinces; spread driven by movement of contaminated pork products.
South Korea	Sporadic	Genotype II	Cases mostly confined to wild boar populations; farm outbreaks remain sporadic and contained.
Thailand	Endemic	Genotype II	Widespread distribution since initial detection in 2022.
Laos	Endemic	Genotype II	High risk of cross-border transmission due to porous borders with endemic neighbors.
Cambodia	Endemic	Genotype II	Sporadic outbreaks continue to be reported.
India	Active Spread	Genotype II	Outbreaks concentrated in Northeast India with signs of spread to other regions.
Indonesia	Regional	Genotype II	Outbreaks reported in major islands including Sumatra and Java.
Malaysia	Regional	Genotype II	Cases present in both Peninsular Malaysia and Borneo (Sabah/Sarawak).

Note: Taiwan’s status has been updated following the WOAH confirmation of the first local outbreak on 25 October 2025. The viral strain detected in Taiwan is highly similar to the genotype I/II recombinant strains currently endemic in China and Northern Vietnam.
